# The Mediating Role of Students' Health Information Literacy Skills: Exploring the Relationship Between Web Resource Utilization and Health Information Evaluation Proficiency

**DOI:** 10.1111/hex.14176

**Published:** 2024-08-15

**Authors:** Umme Habiba, Foujia Sultana Koli

**Affiliations:** ^1^ Institute of Information Sciences Noakhali Science and Technology University Noakhali Bangladesh

**Keywords:** cognition, credible web information, health information, health information literacy, health promotion, online health information, university students

## Abstract

**Background:**

In an increasingly digital age, students rely heavily on web resources to access health information. However, evaluating the reliability and relevance of such information is crucial for informed decision‐making. This study examines the importance of students' health information literacy skills (HILS) as mediators, particularly their ability to utilize web resources and successfully evaluate health information.

**Objectives:**

This research investigates the mediating role of students' HILS in the relationship between their utilization of web resources and their proficiency in evaluating health information.

**Method:**

An online survey was distributed to current students at a public university in Bangladesh as part of the data collection process for this study. Using Google Forms, the authors created a structured questionnaire. The survey was distributed through Messenger groups, Facebook pages and email invitations to reach the target audience effectively. The researchers thoroughly analysed the gathered data using structural equation modelling (SEM) techniques and SmartPLS‐4 software to look for correlations between the variables.

**Result:**

The study revealed that among the 122 participants, a significant number (*N* = 47) accessed internet health information on an occasional basis, whereas 30 individuals reported using it infrequently. The data revealed that 58 individuals, accounting for 47.5% of the sample, possessed the necessary abilities to access and assess online health information. Additionally, 57 participants, representing 46.7% of the sample, demonstrated proficiency in conducting online health information searches. The measurement model demonstrated good convergent validity, as evidenced by composite reliability (CR) scores and Cronbach's *⍺* values over 0.700 and an average extracted variance (AVE) of 0.500. The structural model demonstrated *R* ^2^ values exceeding 0.1, thus validating its dependable forecasting capability. Notable effects were seen, with *f* ^2^ values of 0.335 and 0.317 for the challenges in accessing and evaluating health information (CAEHI) to health information evaluation (HIE) and CAEHI to HILS relationships, respectively. The mediation analysis found that HILS act as a mediator between types of web resources (TWRs) and HIE, with TWR having an indirect impact on HIE through HILS.

**Discussion:**

The result supports all hypotheses. Therefore, it is evident that students' HILS mediate the relationship between utilization of web resources and their proficiency in evaluating health information.

**Conclusion:**

This study's findings could significantly impact instructional practices meant to raise students' health information literacy. This initiative seeks to enable students to make informed decisions about their health by providing them with the necessary tools to analyse and evaluate health‐related information.

**Patient or Public Contribution:**

Research on health information literacy can assist patients and the general public by instructing them on how to assess trustworthy online health resources. Students gave insightful feedback that assisted in shaping the study and guaranteeing its relevancy. If they better comprehend health information literacy, patients and the general public can use web‐based resources and critically evaluate health information more accurately.

## 1 Introduction

Health information literacy (HIL) pertains to an individual's ability to understand health and healthcare information and use it proficiently to make informed decisions and take action about their well‐being. Health literacy (HL) is a vital element of total well‐being, with a substantial impact on community health, safety and the delivery of healthcare services [1]. HIL refers to a range of mastery that allows individuals to discover, understand, analyse and utilize health information to make knowledgeable choices about their health and well‐being [2, 3]. These capabilities allow individuals to efficiently explore extensive information, distinguish reliable sources from disinformation or prejudiced content and ultimately make decisions that enrich their well‐being and those of their communities [4, 5]. HL, as defined by the World Health Organization (WHO), refers to a person's social and cognitive capacities, which include motivation and particular skills needed to achieve, understand and process health information to maintain good health [6, 7]. Proficiency in this skill is essential for effectively searching, comprehending and interpreting health‐related information, which is essential for improving individual health outcomes and the administration of healthcare techniques [8]. By extending this knowledge, students can acquire reliable health information, comprehend its significance and use it to make wise decisions about their health. Because of this empowerment, they may seek appropriate medical attention, take proactive measures and manage their conditions more skilfully. Moreover, it enables administrators and healthcare practitioners to make evidence‐based decisions, improve the standard of care and maximize resources within healthcare organizations [9, 10, 11].

Due to the comprehensive internet usage, many people depend on online platforms to acquire health‐related information. Accessing a wide range of health‐related information online is conceivable, but adequately utilizing these resources requires specialized skills that not all consumers have. Assessing the reliability and accuracy of internet content can be complex, posing challenges in formulating knowledge based on evidence about health matters [12]. Assessing the quality of healthcare resources, using sophisticated search techniques to find appropriate information, assessing the trustworthiness of sources and understanding the advantages and disadvantages of various media formats are necessary for exploring health information [13]. Examining the correctness, relevancy and consistency of the information provided is vital in determining the quality of the resources. In the plethora of data that are available online, advanced search techniques permit people to quickly and effectively acquire specific and targeted health information [14]. Evaluating the dependability of information sources is vital to control misinformation, which can result in incorrect choices about one's health. Knowing the advantages and weaknesses of various media formats—including websites, scholarly journals, films and social media—allows people to choose reliable and the most appropriate sources for their purposes [15]. To make informed judgements concerning one's health and medical treatment, these capabilities are needed in order to successfully explore the broad range of health information that is readily available online [3, 16]. Students' capability to apprehend and utilize health information in educational settings must be prioritized. This emphasis enhances academic achievement, fosters professional development and assists people in better managing their health. Educational institutions may teach students to evaluate health information critically, make knowledgeable health decisions and use this understanding in their personal and professional lives by giving them this proficiency [17]. Furthermore, by encouraging a more knowledgeable and health‐conscious society, this focus on HL can improve people's health outcomes [18]. Providing students with robust HIL abilities helps them navigate a more complicated healthcare environment, foster critical thinking and make well‐informed decisions. Students with these proficiencies are more likely to participate actively in health‐related coursework, contribute to academic discussions and research and facilitate evidence‐based practices [19, 20].

This study intends to evaluate HL levels among Bangladeshi university students and explore how this proficiency mediates the relationship between their usage of online resources and their capacity for determining authentic health information. The study further investigates the correlation between HIL and students' ability to choose trustworthy health information from diverse online resources. It seeks to understand the vital role that HL plays in empowering students to search the comprehensive and sometimes daunting digital environment. This study will shed light on the effectiveness of the current instructional approaches. It may help shape interventions that are used in the future to improve university students' HL. However, acquiring insight into this relationship is crucial for developing educational strategies to enhance students' HIL aptitudes and foster evidence‐based decision‐making within the context of Bangladesh's universities and globally. By understanding the complex relationships between the use of online resources, the capacity to comprehend health information and the decision‐making process, educators can construct specific interventions that successfully qualify students with the necessary skills to navigate the extensive and sometimes intricate realm of health‐related information. These treatments may contain comprehensive instruction in critical thinking, appraisal of internet sources and pragmatic techniques for distinguishing trustworthy health information from dubious sources. By developing these capabilities, students become more equipped to make well‐informed preferences about their well‐being, resulting in improved personal health results. It is also conceivable that using an all‐encompassing approach to health education will improve healthcare practices globally. An educated population with HL is more inclined to participate in proactive health measures, comply with treatment programmes and effectively advocate for their health requirements. Consequently, this may reduce the pressure on healthcare systems, decrease healthcare expenses and enhance the overall standard of treatment. Teachers who integrate HL instruction into their curricula give students more power and assist in constructing a society that is more aware of health issues and progresses towards improved global health practices and legislation.

## 2 Literature Review

Health information refers to any personal data related to an individual's health or disability, including both factual information and subjective viewpoints on illness. According to Alamantariotou [21], it refers to the combination of resources and services that may be accessed through online platforms. These resources and services aim to advocate preserving good health as part of social inclusion programmes. Sabelli et al. [22] highlight the various reasons why health information is required, including enhancing understanding of health needs, helping individuals decide when to seek medical help, choosing suitable healthcare providers and educating patients about general health issues and preventive measures. The Association of College & Research Libraries (ACRL) states that information literacy involves identifying information requirements and utilizing techniques to discover pertinent resources that fulfil those needs. Moreover, credible information is defined by its capacity to substantiate that an event has occurred or will occur, considering its origin and surrounding context. *Credible resources* are defined as reputable sources that offer accurate information. The appraisal of trustworthiness is commonly based on two primary metrics: reliability and knowledge, as emphasized by Habiba and Islam [23] and referenced in Metzger [24]. DeLone and McLean [25] highlight the need to assess the quality of information, especially in terms of timeliness, clarity, relevance, correctness, conciseness, completeness and usability of resources. Incorporating dependable sources in educational research papers is essential as it indicates that claims are substantiated by credible proof. Wertgen and Richter [26] stress the significance of including reliable sources in academic writing to improve the information's legitimacy and strengthen the argument's validity.

### 2.1 Health Information Literacy Skills

In the age of information explosion, it is becoming increasingly more vital to be able to search, understand and apply health information. Proficiency in HIL is paramount for making informed choices about health and overall well‐being [27]. These aptitudes contain a range of mastery, including accessing, analysing and applying effective search techniques for obtaining health‐related information. Given the vast amount of health information available from diverse sources, including traditional media, online platforms and human contacts, possessing vigorous health information literacy skills (HILS) is essential [5, 28]. HIL proficiency helps individuals to navigate through the extensive amount of health information accessible, distinguish reliable sources from inaccurate information and make well‐informed decisions that enhance their own health and the health of their communities. These abilities empower individuals to examine health information discerningly, determine its reliability and pertinence and use it to tackle health‐related issues and make well‐informed preferences regarding healthcare alternatives [29]. Robb and Shellenbarger [30] performed a study to assess undergraduate college students' e‐HL levels, namely, their capacity to obtain, understand and appraise health information from electronic sources. The study results showed that the students had a high level of e‐HL, which was even higher than what was found in previous studies, including high school students and college students specializing in occupational health. The survey participants showed a high level of self‐assurance in their ability to effectively search for, comprehend and evaluate health‐related resources online.

The e‐HL levels of undergraduate college students were examined by Kim and Syn [31] with the expectation that these levels would be particularly higher than average. According to the study's findings, the students' level of e‐HL was higher than that of earlier studies, including college and high school students, with an emphasis on occupational health. The participants indicated high confidence in their capability to find, understand and assess health information from electronic sources. Similarly, university students frequently search for health information on social networking sites on the Internet [32, 33]. According to Senkowski and Branscum [34], search engines are the primary source of Internet health information for undergraduate students, with Google being the most famous. Undergraduate students assess online health information based on visual appeal and ancillary dependability indicators [35]. They repeatedly depend too much on search engine rankings. As a result, undergraduate college students must learn about trustworthy websites about health and expand the critical evaluation skills necessary to determine the calibre of web health information.

### 2.2 Types of Sources that the University Students Used for Accessing Online Health Information

To locate online health information, university students utilize a variety of sources, representing the vast array of platforms available in the contemporary online environment [36, 37]. A study by Ashkanani et al. [38] stated that college students frequently use the Internet to obtain or to locate health‐related information. The study's findings revealed a widespread dependence on web‐based platforms for acquiring health information, as most students acknowledged utilizing the Internet to look for health‐related material. Most importantly, in contrast to younger demographics, the study also indicated a significant increase in online searches for health‐related information across older age cohorts. This indicates that a pattern of internet usage for health‐related questions is developing across various age groups. The survey also demonstrated how typical it is for students to use electronic devices to search for health information online, emphasizing the significant contribution of technology to the ability of college students to look for health information. It is still vital to look into how students specifically utilize social media platforms to acquire and exchange health‐related information, even though utilizing these platforms to locate health information is becoming more widespread. It is necessary to examine social media activities as a source of health information in detail in order to enhance undergraduate students' understanding of substantial health resources [39, 40]. Comparing it to other online and traditional media sources helped to determine its efficacy and trustworthiness. This study can contribute useful knowledge of the mechanisms governing the dissemination and consumption of health information among university students. This can help in designing targeted educational initiatives that facilitate utilizing and critically examining health information from a variety of sources [39].

Furthermore, Maaß et al. [41] asserted that mobile health applications, or ‘health apps’, that are available on tablets and smartphones offer convenient ways to access health data, services and tracking tools. University students can utilize these apps for diverse purposes, such as observing their level of physical fitness, managing chronic health issues and accessing educational resources. College students can obtain trustworthy sources of health information based on scientific evidence on websites run by reputable health organizations and government health agencies like the WHO [5]. Additionally, one can use online communities and forums, like the health‐related subreddits on Reddit and specialized health forums, to request or ask questions, share personal experiences and engage in discussions regarding health‐related subjects with peers and experts [42, 43]. In addition, in the study carried out by Zhang [44], it was discovered that Facebook is a well‐known source of health and well‐being information for college students. However, a recent Pew survey demonstrates that social media sites are not frequently used for health‐related searches. Instead of addressing more severe medical issues, the people who vigorously look for health information on these platforms do so mainly to acquire health updates, advice on lifestyle choices and treatments for minor illnesses. When users' opinions of social networking sites are investigated, concerns regarding the accuracy of the information given and the lack of medical competence among peers are revealed. This study has inherent limitations about the extent and reliability of the data obtained because health information is sensitive.

### 2.3 Evaluation of the Credible Health‐Related Information Bestowed on Online

Evaluating the dependability and authenticity of web‐based health information is important to ensure that individuals acquire accurate, trustworthy and legitimate information to support their decisions regarding their health [45, 46]. Ivanitskaya, Boyle, and Casey [47] set out to assess college‐age individuals' proficiency in locating and evaluating online health resources. The study also aimed to determine if they could specify the difference between peer‐reviewed academic sources. The study also attempted to measure participants' awareness of their level of competency in health information. Through the use of a validated and trustworthy assessment tool known as the research‐readiness self‐evaluation, this study systematically examines adults' health information competency in college. According to a study by Akhtar et al. [48], most participants had a moderate level of e‐HL. These outcomes recommend that participants understand where to obtain useful health resources on the internet and how to use them to address health‐related challenges effectively. According to Robb and Shellenbarger [30] and Hanik and Stellefson [49], information professionals at participating universities should develop and execute a structured curriculum that particularly strives to enhance students' proficiency to locate and evaluate electronic health resources for improving e‐HL among undergraduate students significantly. These studies highlight the importance of assessing and promoting e‐HL among individuals in college, given the growing reliance on e‐resources for health information. By teaching students the necessary abilities to investigate and evaluate online health information critically, universities can empower students to make informed decisions about their well‐being and health [50]. Additionally, implementing well‐structured educational initiatives can guarantee students' proficiency with online health information and greatly enhance their e‐HL abilities.

Zhang [51] demonstrated that there are significant concerns among college students regarding the accuracy of health information, specifically when it comes to social media sites. They emphasize the need for a better understanding and assessment of the validity of health‐related information available on these platforms. Again, students are anxious about how well‐versed their peers are in health‐related social networking sites. Because of this, students are more willing to trust and utilize guidance from individuals who have professional medical experience or who share comparable interests, needs or health concerns. University students might not view the Internet and social media platforms as trustworthy sources of health information, even though they are frequently used for this purpose [52]. This suggests that although these platforms could be valuable information sources, students are reluctant to consider them as accurate, especially regarding health content.

### 2.4 Challenges that University Students Face While Accessing and Evaluating Online Health Information

University students face many challenges while searching for and assessing online health information. These drawbacks are mainly generated by the vast amount of readily available information, variation in the resource's dependability and individual differences in HL. Finding trustworthy sources of online health information presents several challenges for people, according to a study by Colditz et al. [53]. They regularly face overly complex, biased, unreliable information or a mix of these. Furthermore, individuals could use ‘shortcuts’ when looking for information, which could lead to lower quality data being acquired. The potential advantages and drawbacks of teenagers searching for health information online are still mostly unknown. However, putting in place a structured online HL instruction programme has the potential to improve teenagers' proficiency in locating reliable and relevant health information. Al‐Jumaili et al. [54] emphasized in their study the negative consequences that insufficient support systems, such as the scarcity of knowledgeable technical support teams and slow internet connections, have on students' desire to use technology for learning. Furthermore, Hsu [55], cited by Chen and Lee [56], emphasized the necessity for people to comprehend how to discover and assess data from diverse internet sources that have varying degrees of quality. In the research carried out by Zalat, Hamed, and Bolbol [57], numerous impediments were found to utilizing e‐learning tools. Some of these obstacles are a lack of computers and laptops, insufficient computing facilities, inconsistent and limited internet connectivity and other technological issues. These problems indicate how important it is to address technological and infrastructure barriers to ensure that all students have equitable access to online resources. Moreover, it is necessary to raise people's digital literacy and provide structured training on interpreting and evaluating online health information. These steps are required to enable people to analyse and evaluate online health information properly.

## 3 Research Gap

There is increasing research on HIL and its effects on health outcomes, although not much is focused on Bangladesh. Although studies worldwide have emphasized the importance of using web resources and being proficient in evaluating health information, much research remains to be done in Bangladesh on how students' self‐efficacy mediates the relationship between web resource use and online health information evaluation (HIE). Research has been conducted on general HL in Bangladesh, but most of it has not focused on the digital aspect of HIL among students [1, 58, 59, 60, 61, 62, 63, 64, 65, 66]. This distinction is noteworthy because people rely more on digital media to obtain health information, especially younger people who are more willing to use the internet for health‐related searches. Extensive investigation is also needed to explore the mediating role of HIL proficiency and estimate the direct relationship between the usage of online resources and the capability to assess health information. This entails understanding Bangladeshi students' skills or skills that they do not have and how proficiency in these affects their capacity to assess internet health material. Completing this research gap will help policymakers, education instructors and healthcare professionals develop focused interventions that will improve HIL and, in turn, enhance health outcomes in Bangladesh.

## 4 Research Questions

The following queries drove the research:

RQ1. How proficient are university students at searching for health‐related information from online sources?
RQ2. What types of sources do the university students use for accessing online health information?
RQ3. How can university students evaluate credible health‐related information provided online?
RQ4. What types of challenges do university students face while accessing and evaluating online health information?
RQ5. How do HILS mediate the relationship between students' utilization of web resources and their proficiency in evaluating health information techniques?

## 5 Hypotheses

H1. There is a significant impact of types of web resources (TWRs) on HILS.
H2. There is a significant impact of TWRs on students' HIE techniques.
H3. HILS have a significant positive impact on students' HIE techniques.
H4. Students' HILS are significantly influenced by the challenges that students face in accessing and evaluating health information.
H5. Students' HIE techniques are significantly impacted by the challenges that students face in accessing and evaluating health information.
H6. HILS mediate the relationship between students using web resources and HIE techniques.

## 6 Methodology

### 6.1 Research Design

**Figure 1:**
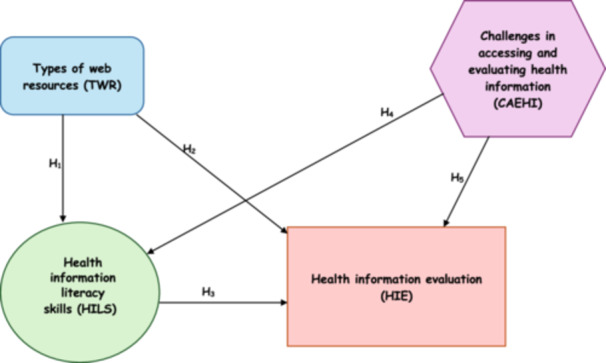
Conceptual framework (self‐developed by the authors).

This cross‐sectional study used a quantitative method to examine how students' HIL proficiency influences the relationship between their usage of online resources and their ability to assess online health information. The authors self‐developed the conceptual framework, which helps as the fundamental baseline for this investigation. Additionally, this framework establishes the theoretical foundations and connections between important variables, guiding the overall research and analysis. Initially, the authors combined existing material to determine important theoretical notions and conceptualize their relationships. The authors self‐developed a framework by following the existing theories that are closely related with, for example, Nutbeam's Health Literacy Framework by Nutbeam [67]; Sørensen et al.'s Integrated Model by Sørensen, Van der Broucke, and Fullam [7]; and Norman and Skinner's [68] eHealth Literacy Model (eHEALS). However, this study framework (see Figure 1) summarizes the expected connections between the studied variables. However, data were gathered by a systematic online survey administered to university students. The survey collected data on three main factors: the frequency and TWRs utilized for health information, the level of skills in HIL and proficiency in evaluating health information strategies. Structural equation modelling (SEM) was used to analyse the data, specifically focusing on the indirect effects of web resource utilization on the ability to evaluate information mediated by HIL abilities. This methodology enables the investigation of both direct and indirect connections, providing a valuable understanding of the possible influence of HIL as a crucial intermediary in the era of digital technology.

### 6.2 Sampling

The selection of sample size and sampling procedure in this research was crucial to guarantee the data's accuracy and reliability. The technique of random sampling, widely used in research, was selected to mitigate bias and guarantee that every individual in the population had an equitable opportunity to be selected for participation. The study focused on students enrolled in a public university. Random sampling is an equitable and impartial approach to selecting participants, which is particularly useful in universities with a potentially big and diverse student population. To enhance the generalizability of the findings, researchers can use random sampling, which helps eliminate bias towards specific groups or individuals [69, 70]. For this study, a sample size of 250 participants was primarily selected and 122 replies were obtained, which is deemed sufficient for several research investigations, particularly when using random sampling. The participants were selected from different departments under various faculties and institutes within the institution, guaranteeing participation across diverse academic subjects and fields of study. This methodology allowed researchers to encompass various viewpoints and encounters about skills in comprehending health information, utilizing online resources and evaluating health information.

### 6.3 Questionnaire Structure

An online version of the questionnaire was designed to gather data from students of different faculties and institutes. The Cronbach alpha (*⍺*) score was obtained to determine the reliability of the questionnaire items [71, 72, 73] used in this study, and it was found that the questionnaire items had a reliable *⍺* score. The questionnaire items were selected from previous studies (see File S1). The questionnaire included the following items:

i. Demographic characteristics such as gender, age, faculty and location and means of gaining access to the Internet;
ii. Frequency of accessing online health information;
iii. Students' proficiency level regarding searching online health information;
iv. Types of sources that the university students used for accessing online health information;
v. Evaluation of the credible health‐related information available online; and
vi. Challenges that university students face while accessing and evaluating online health information.

### 6.4 Data Collection

The data gathering for this study was deliberately carried out over 3 months, specifically from December 2023 to February 2024. The principal approach for gathering data included circulating an online survey through Google Forms to a sample of students chosen randomly from different departments and faculties within the public university. The decision to use this digital method was based on its convenience, cost‐effectiveness and ability to reach a large audience among the target group. The survey was carefully crafted to gather students' perspectives on their HILS and their strategies to evaluate the trustworthiness of health material discovered online. To guarantee the survey's clarity, relevance and accessibility, a crucial initial step was undertaken: the pretesting phase. Before the official commencement of the survey, a preliminary trial was conducted with a select group of students chosen to represent the larger university population accurately. This pretest aimed to assess the clarity, conciseness and impartiality of the questionnaire's questions. The feedback received from this initial group of respondents was important, as it provided significant insights to improve the survey instrument. The input prompted adjustments that aimed to improve the survey's clarity and ease of completion, reducing the likelihood of misunderstandings or confusion among the primary group of participants.

### 6.5 Data Analysis

The analysis method entailed analysing the mediating function of students' HILS in relation to their utilization of web resources and their competency in appraising health information strategies. At first, a heterogeneous group of students was chosen from different educational backgrounds. A thorough survey instrument was created, which included validated scores to assess HILS, habits of using web resources and proficiency in evaluating health information strategies. After conducting pilot testing to improve the survey, data were gathered from participants to ensure both clarity and anonymity. Descriptive analysis was carried out to calculate statistical measures to describe the sample and the responses. After gathering the data, researchers performed SEM analysis to evaluate the proposed hypotheses. Researchers used SmartPLS to rigorously verify the validity and reliability of measurement models and investigate the structural relationships between latent variables. The measurement scale's reliability was assessed using composite reliability (CR) coefficients and Cronbach's *⍺* metrics. Afterwards, the study's validity was carefully examined by evaluating discriminant validity. This process involved comparing the average variance extracted (AVE) with inter‐construct correlations.

Furthermore, the AVE was utilized to evaluate convergent validity. Additionally, a thorough analysis of item loadings and cross‐loadings was performed to verify that the items accurately assessed the intended constructions. SmartPLS enabled the examination of the structural model (SM) by utilizing path coefficients, goodness‐of‐fit indices, effect size (*f* ^2^) and predictive relevance (*R* ^2^). This methodology yields accurate estimations, especially for intricate models that incorporate hidden variables and have restricted sample sizes, even when dealing with atypical data distributions [67, 74, 76]. The study assessed the path coefficients, significance thresholds and impact sizes to ascertain the direction and magnitude of the relationships between variables. This thorough examination improves the trustworthiness and accuracy of the results by providing a more profound comprehension of the connections within the model.

## 7 Results

### 7.1 Students' Demographics and Academic Information

Table 1 presents the participants' demographic information (*N* = 122), including their gender, age, academic status, geographical location, affiliation with a faculty or institute and educational level. Of these, 77 (63.1%) were men and 45 (36.9%) were women. Eighteen (14.8%) were between 16 and 20 years of age, whereas 99 (81.1%) were between 21 and 25 years of age. The Institute of Information Sciences showed the highest rate of participation, with *N* = 69 replies, followed by the Faculty of Science, with *N* = 14. There were 16 postgraduate participants, and *N* = 106 were undergraduate students. In terms of location of residence, *N* = 38 lived in rural areas and *N* = 84 lived in urban areas. In terms of using the Internet to obtain health information, 22 people used it regularly, *N* = 30 people utilized it seldom and *N* = 47 people accessed it occasionally. Furthermore, *N* = 24 students especially comprehended the term ‘HIL’, whereas *N* = 22 students did not know what this term was. Of the *N* = 76 students, 62.3% were familiar with this term.

**Table 1:** Students' demographics and academic information.

Demographic characteristics	Categories	No. of participants (*n* = 122)	%
Gender	Female	45	36.9
	Male	77	63.1
Age (in years)	16–20	18	14.8
	21–25	99	81.1
	26 and above	5	4.1
Academic discipline	Faculty of Engineering and Technology	4	3.3
	Faculty of Science	14	11.5
	Faculty of Business Administration	4	3.3
	Faculty of Social Science and Humanities	15	12.3
	Faculty of Education Science	8	6.6
	Faculty of Law	2	1.6
	Institute of Information Sciences	70	57.4
	Institute of Information Technology	5	4.1
Educational level	Undergraduate	106	86.9
	Postgraduate	16	13.1
Location of residence	Urban area	84	68.9
	Rural area	38	31.1
Frequency of use of online platforms for accessing health information	Almost always	19	15.6
	Very often	22	18.0
	Sometimes	47	38.5
	Rarely	30	24.6
	Never	4	3.3
Comprehension of the term ‘health information literacy’	Yes	76	62.3
	No	24	19.7
	Unsure	22	18.0

### 7.2 Measurement Model Assessment

Evaluation of the validity and reliability of the measurement tools used to operationalize latent components is a crucial step in SEM, referred to as measurement model assessment. This evaluation seeks to verify the alignment of the measurement model with the theoretical concepts that it represents. It does so by analysing the relationships between observed variables and the corresponding latent constructs, as highlighted by Ahmad, Zulkurnain, and Khairushalimi [77], Janadari et al. [78] and Hair, Hult, and Ringle [75]. Table 2 was used to examine the reliability of variables throughout the evaluation of the measurement model, using both Cronbach's *⍺* and CR. Notably, all CR scores surpassed the criterion of 0.700, as Wasko and Faraj [79] recommended. Furthermore, Cronbach's *⍺* values for each construct were above the criterion of 0.700, indicating that the internal consistency was good. Moreover, the computed average extracted variance (AVE) was found to be higher than 0.500, indicating that all the constructs have convergent validity. This suggests that the observable variables efficiently align with their corresponding underlying constructions.

**Table 2:** Factor loadings, reliability and validity.

Construct	Item	Loading	Cronbach's *⍺*	rho_*A*	CR	AVE	VIF
HILS			0.940	0.944	0.949	0.675	
	HILS1	0.779					2.418
	HILS2	0.824					2.971
	HILS3	0.802					3.305
	HILS4	0.848					4.239
	HILS5	0.815					2.885
	HILS6	0.823					3.089
	HILS7	0.843					3.039
	HILS8	0.869					3.325
	HILS9	0.789					2.563
TWR			0.877	0.909	0.901	0.515	
	TWR1	0.440					1.609
	TWR2	0.446					1.893
	TWR3	0.585					2.273
	TWR4	0.789					2.186
	TWR5	0.722					1.875
	TWR6	0.854					2.958
	TWR7	0.823					2.661
	TWR8	0.811					2.694
	TWR9	0.833					2.453
HIE			0.932	0.933	0.944	0.678	
	HIE1	0.873					3.645
	HIE2	0.825					3.261
	HIE3	0.833					2.658
	HIE4	0.814					2.383
	HIE5	0.809					2.481
	HIE6	0.853					3.200
	HIE7	0.776					2.282
	HIE8	0.800					2.231
LAP			0.911	0.917	0.928	0.617	
	CAEHI1	0.761					2.276
	CAEHI2	0.814					2.919
	CAEHI3	0.834					3.030
	CAEHI4	0.833					3.242
	CAEHI5	0.787					2.121
	CAEHI6	0.806					2.629
	CAEHI7	0.737					2.739
	CAEHI8	0.701					2.170

Abbreviations: AVE, average variance extracted; CR, composite reliability; HIE, health information evaluation; HILS, health information literacy skills; LAP, learning and academic performance; TWR, types of web resources.

Table 2 provides a comprehensive summary of the validity and reliability evaluations, including the factor loadings for each item. These assessments are essential for assuring the strength and accuracy of the measurement model. In addition, a thorough discriminant validity assessment was performed using the Heterotrait–Monotrait Method (HTMT), as per the criteria set by Fornell and Larcker [80], as outlined in Table 3. This methodological approach helps ascertain the distinctiveness of measurements, which is crucial for ensuring the uniqueness of the studied constructs. Furthermore, a thorough analysis of multicollinearity issues was conducted to evaluate the existence of any problems. It was determined that all indicator variance inflation factors (VIFs) were below the commonly accepted threshold of 5, demonstrating the absence of serious issues with multicollinearity.

**Table 3:** Discriminant validity using the criterion of Fornell and Larcker and the HTMT.

	CAEHI	HIE	HILS	TWR
HTMT				
CAEHI				
HIE	0.817			
HILS	0.673	0.733		
TWR	0.587	0.658	0.548	
Fornell and Larcker				
CAEHI	0.785			
HIE	0.761	0.823		
HILS	0.634	0.693	0.822	
TWR	0.558	0.628	0.509	0.718

Abbreviation: HTMT, Heterotrait–Monotrait Method.

**Figure 2:**
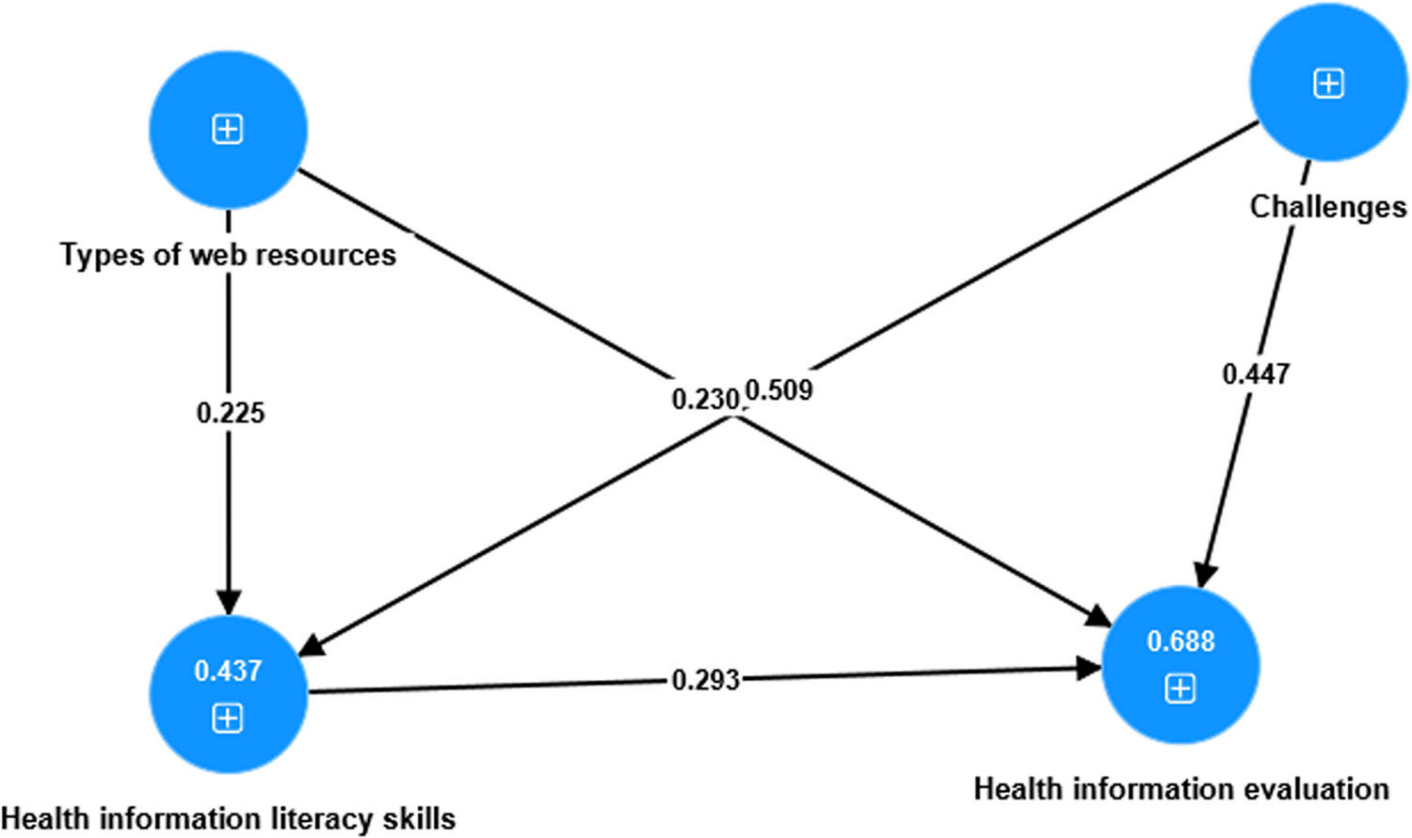
Measurement model assessment.

Moreover, Table 4 presents extensive data regarding the cross‐factor loadings of each individual item. Significantly, every factor loading surpassed its corresponding cross‐loading, except for three items (TWR1 *λ* 0.440, TWR2 *λ* 0.446 and TWR3 *λ* 0.585). This demonstrates robust discriminant validity among the constructs, suggesting that the measurements efficiently differentiate between the underlying constructs. These comprehensive assessments collectively guarantee the measurement model's dependability, precision and ability to differentiate, establishing a strong basis for further analyses and interpretations. Additionally, see also Figure 2 for details about measurement model assessment.

**Table 4:** Discriminant validity—Cross/factor loadings.

	CAEHI	HIE	HILS	TWR
CAEHI1	0.761	0.586	0.416	0.363
CAEHI2	0.814	0.686	0.548	0.476
CAEHI3	0.834	0.646	0.491	0.471
CAEHI4	0.833	0.692	0.539	0.454
CAEHI5	0.787	0.598	0.49	0.431
CAEHI6	0.806	0.601	0.577	0.489
CAEHI7	0.737	0.492	0.472	0.452
CAEHI8	0.701	0.425	0.43	0.349
HIE1	0.654	0.873	0.598	0.568
HIE2	0.667	0.825	0.558	0.502
HIE3	0.632	0.833	0.575	0.552
HIE4	0.564	0.814	0.606	0.542
HIE5	0.636	0.809	0.627	0.497
HIE6	0.616	0.853	0.526	0.488
HIE7	0.602	0.776	0.516	0.465
HIE8	0.637	0.800	0.552	0.518
HILS1	0.626	0.574	0.779	0.446
HILS2	0.507	0.576	0.824	0.419
HILS3	0.402	0.489	0.802	0.403
HILS4	0.468	0.566	0.848	0.436
HILS5	0.503	0.475	0.815	0.391
HILS6	0.557	0.615	0.823	0.389
HILS7	0.479	0.571	0.843	0.363
HILS8	0.646	0.688	0.869	0.489
HILS9	0.436	0.520	0.789	0.404
TWR1	0.116	0.241	0.243	0.440
TWR2	0.174	0.186	0.229	0.446
TWR3	0.251	0.244	0.312	0.585
TWR4	0.500	0.528	0.393	0.789
TWR5	0.417	0.479	0.349	0.722
TWR6	0.401	0.444	0.337	0.854
TWR7	0.455	0.531	0.417	0.823
TWR8	0.578	0.620	0.464	0.811
TWR9	0.457	0.534	0.451	0.833

### 7.3 SM Assessment

The SM was evaluated with great attention to detail, with the goal of clarifying certain crucial metrics vital for comprehending the relationships within the researched framework. The metrics used in the analysis were the coefficient of determination (*R*‐squared, *R* ^2^), predictive relevance (*Q* ^2^) of exogenous influences on endogenous ones, effect sizes (*f* ^2^), path coefficients (*β*) and their corresponding statistical significance. The coefficient of determination (*R* ^2^) quantifies the extent to which the model accounts for the variability in the dependent variable. This statistic is crucial for comprehending the predictive efficacy of the model. Moreover, the *Q* ^2^ value of exogenous factors on endogenous ones offers valuable information about the model's capacity to anticipate unknown data. The assessment procedure also involved careful examination of effect sizes (*f* ^2^) and route coefficients (*β*). Effect sizes quantify the practical importance of the connections between variables, whereas path coefficients clarify the magnitude and direction of these connections. The statistical significance of these coefficients confirms their usefulness in the model. Afterwards, standardized path coefficients were used to assess the theoretical pathways in the study framework. This approach enhances comprehension of the interplay and impact of different elements in the model. It is important to mention that each structural method greatly improved the model's overall quality, as reported by Gallardo‐Vázquez and Sánchez‐Hernández [81]. Evaluating the SM entails thoroughly examining the connections between fundamental constructs and confirming their congruence with empirical evidence and theoretical frameworks. The importance and applicability of path coefficients were predominantly evaluated using metrics such as *R* ^2^ values for endogenous variables, as Briones et al. [82] suggested. For this study, we used a suggested criterion of 0.1, as proposed by Falk and Miller [83]. After examining Table 5, it is evident that the majority of *R* ^2 ^values reach or surpass the 0.1 threshold, showing a noteworthy and strong predictive capacity for specific variables, such as HIE (*R* ^2^ = 0.688) and HILS (*R* ^2^ = 0.437). This discovery is consistent with the suggestions made by Cohen [84], Chin [85] and Hair, Ringle, and Sarstedt [86], 2011, confirming the strength of the model's ability to make accurate predictions.

**Table 5:** Testing direct relationships.

	Original sample (O)	SD	*t* value (bootstrap)	*p* values	BI [2.5%; 97.5%]	Result
H_1_: TWR ‐> HILS	0.225	0.086	2.628	0.009	[0.052; 0.393]	H_1_ is supported
H_2_: TWR ‐> HIE	0.230	0.066	3.500	0.000	[0.102; 0.356]	H_2_ is supported
H_3_: HILS ‐> HIE	0.293	0.089	3.277	0.001	[0.129; 0.480]	H_3_ is supported
H_4_: CAEHI ‐> HILS	0.509	0.080	6.352	0.000	[0.343; 0.655]	H_4_ is supported
H_5_: CAEHI ‐> HIE	0.447	0.085	5.244	0.000	[0.280; 0.615]	H_5_ is supported
*R* ^2^ HIE = 0.688	*Q* ^2^ HIE = 0.620					
*R* ^2^ HILS = 0.437	*Q* ^2^ HILS = 0.405					

Abbreviations: BI, bias‐corrected confidence interval; CAEHI, challenges in accessing and evaluating health information; HIE, health information evaluation; HILS, health information literacy skills; TWR, types of web resources.

**Figure 3:**
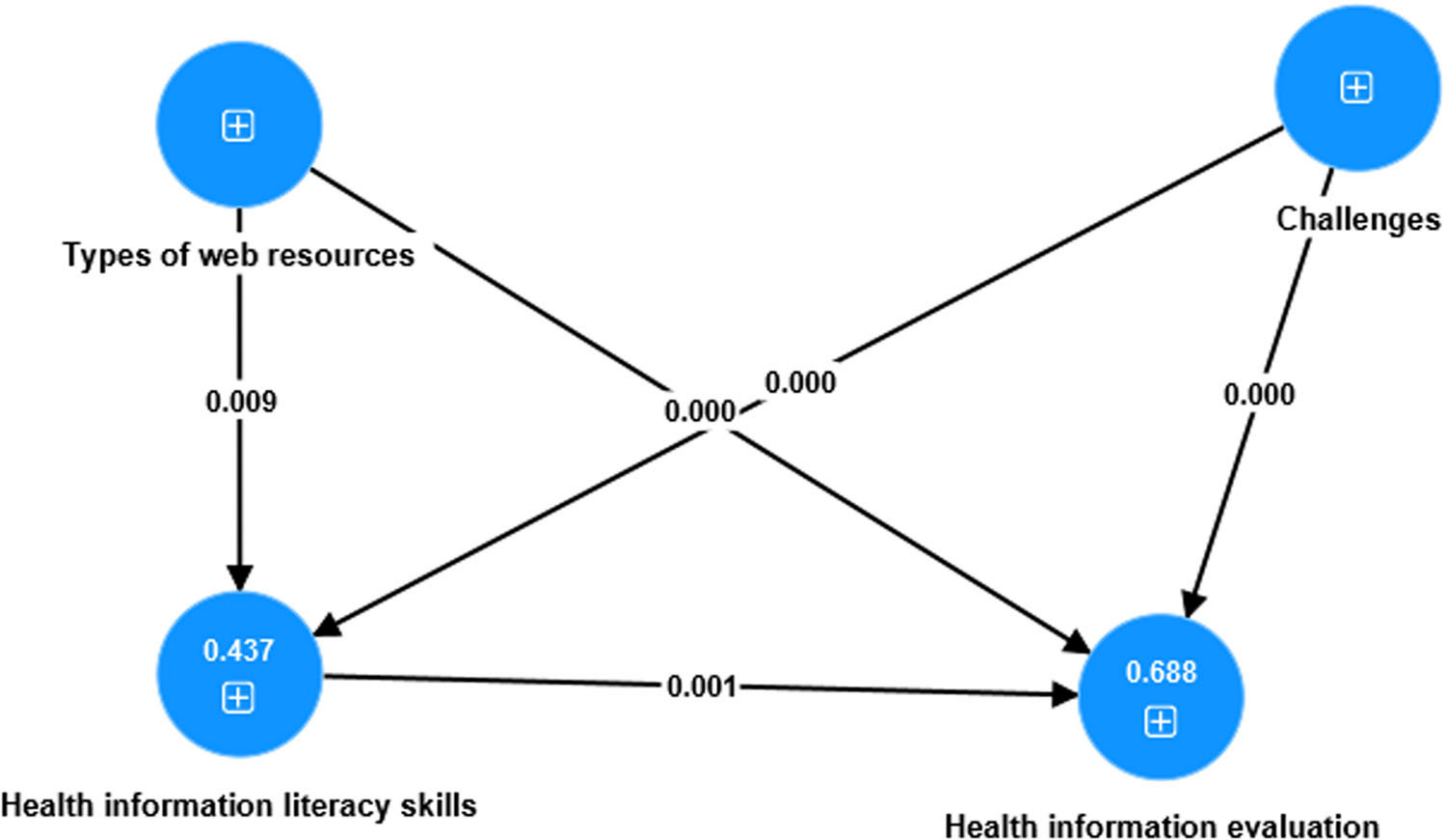
Structural model assessment.

Moreover, the analysis of *Q* ^2^ values provides a more profound understanding of the predictive significance of internal constructs in the SM. *Q* ^2^ values greater than 0.1 are considered important markers of predictive importance. For example, *Q* ^2^ values such as *Q* ^2^ HIE = 0.620 and *Q* ^2^ HILS = 0.405 indicate strong predictive relevance, indicating significant predictive ability for the corresponding constructs. To provide further background information, Hair, Ringle, and Sarstedt [86] have developed a set of standards for evaluating *Q* ^2^ results. Based on these criteria, values of 0.02, 0.15 and 0.35 indicate different degrees of predictive importance. More precisely, a value of 0.02 is considered weak, 0.15 is classified as moderate and 0.35 is described as high for each effect. It is important to note that a *Q* ^2^ value greater than zero indicates that there is predictive relevance in the model. The results shown in Table 5 and Figure 3 highlight the overall importance of the SM's predictive skills, confirming its effectiveness in predicting outcomes.

The assumptions were carefully examined to ascertain the importance of the connections in order to evaluate the suitability of the model comprehensively. The study revealed strong evidence for H_1_, which sought to clarify the significant influence of different TWRs on HILS. The study showed a strong correlation between TWR and HILS (*β* = 0.225, *t* = 2.628, *p* = 0.009), offering strong evidence in favour of H_1_. Hypothesis 2 aimed to determine how much TWR impacted students' appraisal of health information (HIE). The findings revealed a significant correlation, demonstrating that TWR significantly influences HIE (*β* = 0.230, *t* = 3.500, *p* = 0.000), hence confirming the validity of H_2_. In the case of Hypothesis 3, which investigates the impact of students' HILS on their HIE strategies, the results again supported H_3_ by providing evidence that HILS significantly impacted HIE (*β* = 0.293, *t* = 3.277, *p* = 0.001).

Similarly, Hypothesis 4 sought to investigate the possible influence of students' challenges in accessing and evaluating health information (CAEHI) on their HILS. The investigation revealed a substantial impact (*β* = 0.509, *t* = 6.352, *p* = 0.000), supporting Hypothesis H_4_. In addition, the analysis showed significant results about the impact of CAEHI on students' procedures for evaluating health information, referred to as CAEHI ‐> HIE (*β* = 0.447, *t* = 5.244, *p* = 0.000), hence verifying Hypothesis 5. Therefore, the careful examination of hypotheses yielded strong evidence for the connections in the model, confirming the importance of the interactions between the studied variables.

### 7.4 Effect Size *f* ^2^

By eliminating a particular external element from the SM, the author measures its effect size, referred to as *f* ^2^, which reveals its impact on the internal factors within the model [87]. Cohen [88] states that the *f* ^2^ values indicate the intensity of these influences. An *f* ^2^ value of 0.02 indicates a very small impact, but a value between 0.02 and 0.15 indicates a moderate effect. Values exceeding 0.15 suggest a substantial impact. The study found significant effects, with *f* ^2^ values of 0.335 and 0.317 for the routes from CAEHI ‐> HIE and CAEHI ‐> HILS, respectively. These data demonstrate a substantial effect size, indicating a strong impact of the external factor CAEHI on the internal factors HIE and HILS. Subsequently, moderate impacts were observed, with *f* ^2^ values of 0.154 and 0.110 for the routes from HILS ‐> HIE and TWR ‐> HIE, respectively. In summary, the results presented in Table 6 demonstrate different levels of influence among the components in the structural model. The impacts vary in magnitude from significant to moderate, with a complete understanding of their relationships.

**Table 6:** Effect size *f* ^2^.

	*f* ^2^	Effect size
CAEHI ‐> HIE	0.335	Large
CAEHI ‐> HILS	0.317	Large
HILS ‐> HIE	0.154	Medium
TWR ‐> HIE	0.110	Medium
TWR ‐> HILS	0.062	Small

### 7.5 Goodness of Fit

Goodness of fit is a statistical term that evaluates the degree to which a model accurately matches the observed data. It measures the difference between the observed data and the predicted values from the model. Researchers can use it to assess the suitability of their models and discern whether any differences are caused by chance or whether they suggest a substantial lack of compatibility [89, 90]. Assessing the adequacy of the fit is crucial in determining if measurement instruments demonstrate convergent validity, a vital component of SEM [91] emphasizes the need to use appropriate criteria to measure the level of agreement between observed and predicted values, as highlighted by Mérigot, Durbec, and Gaertner [92].

Table 7 presents essential metrics for evaluating the model's adequacy, such as *χ* ^2^ values, a non‐match index (NMI) value and a standardized root mean square (SRMR) value. These indicators are essential for assessing the model's suitability to the data. The *χ* ^2^ value of 999.669, the NFI value of 0.724 and the SRMR value of 0.075 were evaluated by comparing them to the recommended minimum threshold of 0.85 [93]. Remarkably, these values are lower than the necessary threshold, suggesting significant concurrence between the observed data and the anticipated model.

**Table 7:** Goodness of fit.

Model fit	Saturated model	Estimated model
SRMR	0.075	0.075
d_ULS	3.338	3.338
d_G	1.697	1.697
Chi‐square	999.669	999.669
NFI	0.724	0.724

### 7.6 Mediation Analysis

**Figure 4:**
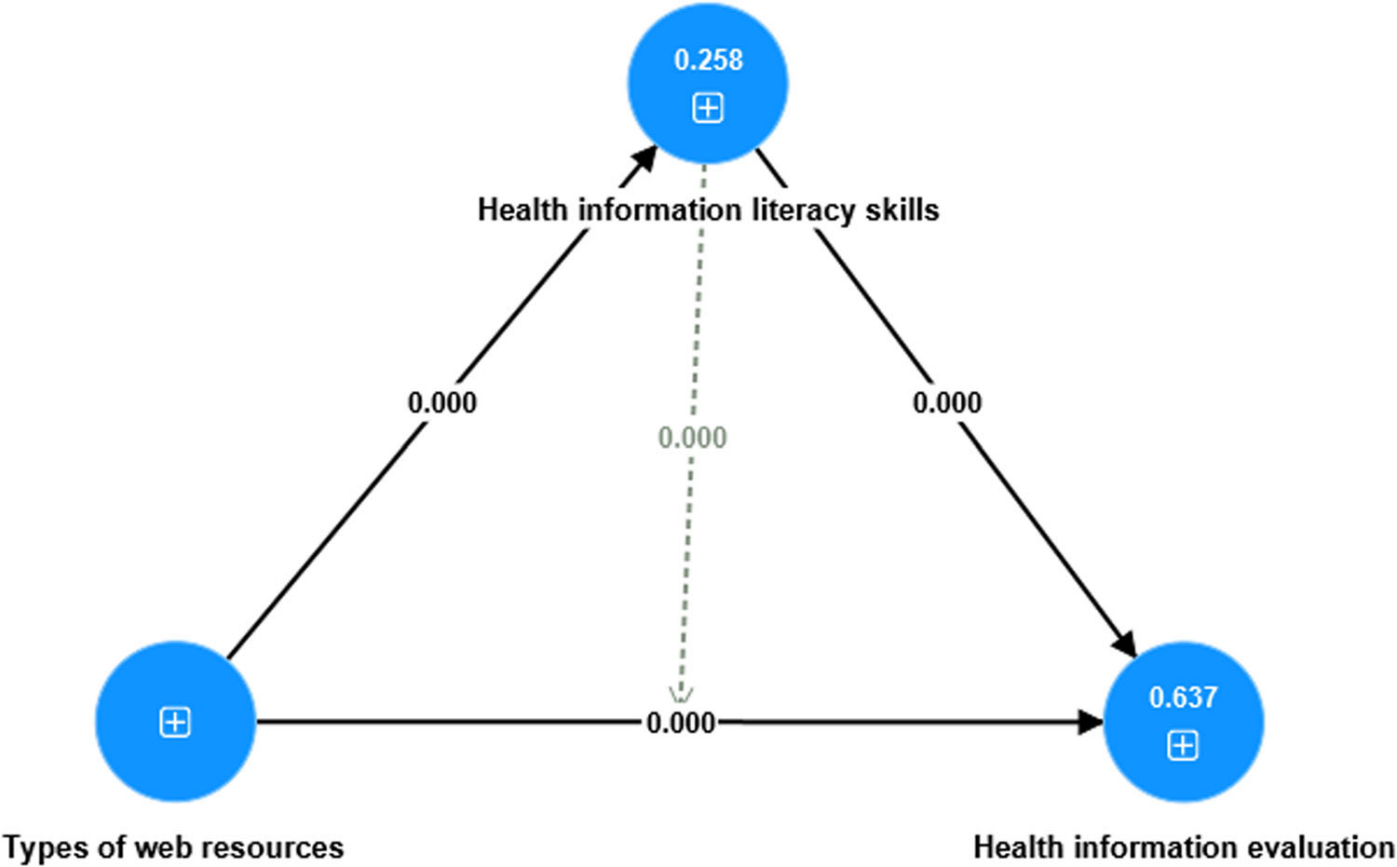
Mediation analysis.

A mediation analysis was performed to investigate the possible impact of students' HILS on the connection between various types of web‐based resources (TWR) and HIE methodologies. As shown in Table 8 and Figure 4, the results provide an essential understanding of this complex interaction. The initial observation revealed that TWR had a substantial and statistically significant impact on HIE (H_6_: *β* = 0.536, *t* = 8.621, *p* < 0.001). When the mediating variable HILS was included in the analysis, the impact of TWR on HIE remained statistically significant (*β* = 0.317, *t* = 4.399, *p* < 0.001). Moreover, we found significant results when investigating the indirect impact of TWR on HIE through HILS, as indicated by the association TWR ‐> HILS ‐> HIE (*β* = 0.219, *t* = 3.811, *p* < 0.001). These data indicate that HILS regulates the connection between TWR and HIE. Therefore, the analysis presents strong evidence supporting hypothesis H_6_, suggesting that HILS is a mediator in this relationship with TWR and HIE.

**Table 8:** Mediation analysis.

Total effects (TWR ‐> HIE)			Direct effects (TWR ‐> HIE)			Indirect effects of TWR on HIE						
Coefficient	*t* value	*p* value	Coefficient	*t* value	*p* value	Hypothesis	Coefficient	SD	*t* value	*p* value	Percentile bootstrap 95% confidence interval	
											Lower	Upper
0.536	8.621	0.000	0.317	4.399	0.000	**H**_**6**_ **: TWR ‐**> **HILS ‐**> **HIE**	0.219	0.058	3.811	0.000	0.122	0.344

Abbreviations: HIE, health information evaluation; HILS, health information literacy skills; TWR, types of web‐based resources.

## 8 Discussions

The study emphasizes the importance of students' HIL proficiency in affecting their capacity to assess health‐related content in an era characterized by the widespread availability of internet information. By explaining the significance of HIL skills in mediating the relationship between web resource utilization and information assessment proficiency, this study presents valuable insights into how educational interventions enable students to become critical consumers of health information. To foster informed decision‐making, enhance health outcomes and generate a more health‐literate society, it is essential to prioritize the development of HIL skills among students. Therefore, educators, policymakers and healthcare stakeholders must prioritize tackling the intricacies of HIL. However, the study's findings suggest that most students depend on internet platforms to obtain health information. In particular, 122 respondents to the study stated that they had used internet resources for this reason. Of these participants, 47 said that they utilize online health information occasionally, whereas 30 mentioned that they rarely have access to it. In addition, a total of 22 students reported regularly receiving health information through the Internet. The present study results align with earlier research; for instance, it indicates that college students use diverse resources to learn about health issues, with the Internet as the main source [91, 94, 95]. Despite its frequent use, people usually consider the Internet as less trustworthy than other sources [52]. Friends, parents, educators, health instructors and health centre workers are considered more reliable and trustworthy sources [52, 96]. Students' usage of sources is also impacted by demographic factors such as age, gender and ethnicity [96]. According to these results, educational institutions ought to utilize widely used platforms to provide reliable health information to students successfully and perhaps change their behaviour in terms of health [51, 52].

Participants were also asked to clarify their understanding of the term ‘HIL’. The findings indicated that 76 students, accounting for 62.3% of the total respondents, were aware of the concept of HIL. Out of the individuals who were familiar with the term, a total of 24 students specifically indicated that they were aware of the phrase. On the other hand, 22 respondents declared that they were unaware of the term ‘HIL’. This study's results match those of Mahmoudi and Taheri [97], who discovered a strong positive association between gender and students about their HL levels. According to Taheri et al. [98], information literacy is a predictor of HL, accounting for 35.4% of its variance. Furthermore, a recent study by Ranjbaran, Chollou, and Babazadeh [99] and Rezakhani Moghaddam, Ranjbaran, and Babazadeh [100] claimed that self‐efficacy was the biggest predictor of preventive behaviours, and they also discovered a substantial link between HL and demographic characteristics, including age, gender and educational level. According to Ivanitskaya, Boyle, and Casey [47], most students believe that they are good or great researchers, though many have problems performing sophisticated searches for information, assessing websites dealing with health issues and identifying reliable sources of information. In a subsequent investigation, Ivanitskaya et al. [101] stated that students preferred self‐directed learning and librarian support and intended to develop their library, Internet and information evaluation skills. These results emphasize the importance of information literacy skill development in improving students' HL and highlight the necessity of focused interventions and resources in academic contexts.

In addition, this study utilized SEM analysis to examine the six hypotheses in detail. The researchers conducted a thorough investigation that encompassed the measurement model, the structural model and mediation analysis. After assessing the measurement model, it was noted that all CR ratings surpassed the acceptable criterion of 0.700. Furthermore, Cronbach's *⍺* values for each construct exceeded the threshold of 0.700, indicating good internal consistency. In addition, the calculated AVE exceeded 0.500, indicating that there is convergent validity among all constructs. This indicates that the observable variables accurately capture the fundamental concepts that they represent. In addition, a thorough evaluation of concerns related to multicollinearity was performed. All indicator VIFs were found to be below the widely accepted threshold of 5, demonstrating the absence of substantial multicollinearity concerns. Significantly, the cross‐factor loadings of each item were higher than their corresponding cross‐loadings, except for three items (TWR1 *λ* 0.440, TWR2 *λ* 0.446 and TWR3 *λ* 0.585).

The structural model assessment indicated that most of the *R* ^2^ values surpass the 0.1 criterion, demonstrating significant and reliable prediction power for some variables, particularly HIE (*R* ^2^ = 0.688) and HILS (*R* ^2^ = 0.437). Moreover, examining the *Q* ^2^ values offers a more profound understanding of the predictive importance of internal constructs in the structural model. *Q* ^2^ values greater than 0.1 are considered important markers of predictive importance. For example, *Q* ^2^ values such as *Q* ^2^ HIE = 0.620 and *Q* ^2^ HILS = 0.405 indicate a high level of predictive relevance, highlighting the significant ability to predict the relevant constructs. In addition, the study thoroughly examined the assumptions that support the model to assess the importance of the linkages, thus ensuring an in‐depth examination of its appropriateness. The study aims to determine the reliability and validity of the proposed model in capturing the intricate connections between students' HILS, utilization of web resources and competency in evaluating health information. The study's findings provided persuasive evidence supporting Hypotheses 1–5 (H_1_–H_5_), demonstrating a robust and statistically significant influence of each hypothesis on the overall model. These findings emphasize the significance of each assumed connection within the conceptual framework, confirming the accuracy of the theoretical statements proposed by the study. The study specifically offered empirical evidence to support the hypotheses suggesting that HIL abilities mediate the connection between the utilization of web resources and proficiency in evaluating health information. The result indicated that HILS is a mediator in this relationship with TWR and HIE. The results of this study are in line with those of Keshavaraz [102], who found that information literacy competences played a mediating role in confirming the indirect impact personal variables on knowledge‐sharing behaviour, with a path coefficient of 0.02. Additionally, Meppelink et al. [103] discovered that cognitive load and ease of imagining mediated the influence of HL on knowledge recall and attitudes towards websites, although website involvement had a negligible effect. Besides, according to Hsu, Chiang, and Yang [104], schools should work to improve students' e‐HL and encourage health behaviours to help them achieve high levels of critical e‐HL. This is because e‐HL is thought to mediate the relationship between individual variables and health behaviours. The current study is also consistent with that of Niu, Willoughby, and Zhou [105], who reported that health‐related social media use and HL influenced health‐related behavioural intentions on social media through their previous effects on self‐efficacy. Self‐efficacy and HL were more closely linked in younger participants.

## 9 Conclusion

In conclusion, the proficiency of students' HIL l skills is crucial in navigating, incorporating and utilizing health‐related information effectively. This study emphasizes how crucial it is to help students develop HIL skills at a time when digital material is widely available. By providing students with the essential skills to assess health information's trustworthiness, significance and practicality, policymakers and educators can enable them to make knowledgeable choices regarding their health and well‐being [106]. Moreover, the results of this study highlight the complex and diverse characteristics of HIL skills, which include the capability to find and retrieve information and the ability to evaluate and understand it critically. To mitigate the risks of misinformation and effectively navigate the complexities of the digital world, students must develop strong HILS. This is particularly important as students increasingly rely on online sources for health‐related information [107, 108]. Beyond academics, the study's ramifications are also noteworthy, as they provide insightful information to educators, legislators and healthcare professionals. Stakeholders should prioritize activities to enhance HL among students by acknowledging the crucial role of HILS in supporting informed decision‐making and improving health outcomes. By promoting a generation of discerning consumers of health information, we can provide individuals with the ability to make proactive decisions about their health, which will contribute to society's overall well‐being. Therefore, as we continue to deal with the challenges of the digital era, it is crucial to invest in initiatives promoting strong HIL abilities. This is essential for creating a more knowledgeable and informed population regarding health matters.

A fundamental strength of investigating the mediating role of students' HILS in the relationship between web resource usage and HIE mastery is that it emphasizes the essential significance of literacy skills in effectively exploring and assessing online health information. In Bangladesh, this is the first time such an attempt has been undertaken. The outcomes of this study can assist in shaping educational strategies and regulations that will enhance students' ability to utilize this proficiency to make knowledgeable health decisions. However, the limited responses, varying digital access and sociodemographic backgrounds of the students could be weaknesses that impact how broadly the results can be applied. Besides, various assessment tools may be needed to accurately estimate HIL and evaluation proficiency, as both tasks might be complicated in Bangladesh. Future studies should examine comprehensively at the university, college and school levels how students' HIL capabilities mediate the relationship between their use of online resources and their proficiency in evaluating health information. Moreover, more extensive research is also necessary worldwide to comprehend the students' and general population's HL proficiency in utilizing web resources and how students assess authentic health information. This entails looking into educational programmes that improve these skills, determining what influences their growth and evaluating the effects of digital access and sociodemographic characteristics. Comprehending these processes will facilitate the development of focused initiatives to enhance HL among students spanning several academic phases.

## 10 Research Limitations

One limitation of this study is its restricted emphasis on a single public university in Bangladesh. To improve the thoroughness and applicability of the research, it would be advantageous to include a wider range of students from different universities around Bangladesh. By including various universities in the study, a more comprehensive and representative sample may be obtained, leading to insights that are more relevant and can be applied to a wider population of students in the country.

## Author Contributions

**Umme Habiba:** supervision, project administration, writing–review and editing, software, validation. **Foujia Sultana Koli:** conceptualization, methodology, data curation, formal analysis, writing–original draft, investigation.

## Conflicts of Interest

The authors declare no conflicts of interest.

## Supporting information

Supporting information.

## Data Availability

The data produced and examined during the present investigation can be obtained upon reasonable request. To obtain access to the data, please get in touch with the respective author. We will try to immediately disclose the data in compliance with relevant data protection legislation.
