# Supplementary material for: The Mediating Role of Students' Health Information Literacy Skills: Exploring the Relationship Between Web Resource Utilization and Health Information Evaluation Proficiency
Source: Health Expect. 2024 Aug 15;27(4):e14176. doi: 10.1111/hex.14176 (PMC11327112; doi:10.1111/hex.14176)
Supplement: Supplementary file 1 — Supporting information. [file HEX-27-e14176-s001.docx]

## **Appendix**

**Survey Questionnaire**

**On**

**The role of students' health information literacy skills as a mediator between the use of web resources and the ability to evaluate health information techniques**

**Section 1: Demographic information**

1. **Gender**


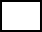
 Female Male
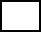


1. **Age (Years)**

| 16-20 | 21-25 | 26 and above |
| --- | --- | --- |

1. **Faculty/Institute name for university students. (For University students)**

| Faculty of Engineering and Technology |
| --- |
| Faculty of Science |
| Faculty of Business Administration |
| Faculty of Social Science and Humanities |
| Faculty of Education Science |
| Faculty of Law |
| Institute of Information Sciences |
| Institute of Information Technology |

1. **Level of study**

| Undergraduate | Postgraduate |
| --- | --- |

1. **Where are you located now?**

| Urban Area | Rural Area |
| --- | --- |

1. **How do you get access to the Internet at your residence? (Check all that apply)**

| Mobile Operator data package | Wi-Fi | I do not have internet at home |
| --- | --- | --- |

1. **How often do you use online platforms for accessing health information?**

| Almost Always | Very Often | Sometimes | Rarely | Never |
| --- | --- | --- | --- | --- |

**Section 2: Health Information literacy skills among college and university students**

1. **Do you know what health information literacy is?**

| Yes |
| --- |
| No |
| Unsure |

1. **How proficient are university students at searching for health-related information?**

Please indicate your agreement or disagreement with each of the following statements about the health information literacy skills that you have-

| **Health information literacy skills (HILS)** | **Strongly**  **disagree** | **Disagree** | **Undecided** | **Agree** | **Strongly**  **agree** |
| --- | --- | --- | --- | --- | --- |
| I can incorporate concepts from  consulted health information resources |  |  |  |  |  |
| I can organize the health information to locate the main  ideas, key issues, etc. |  |  |  |  |  |
| I know the information retrieval techniques to collect health information from the Internet |  |  |  |  |  |
| I have the skills to evaluate the health information available on  the Internet |  |  |  |  |  |
| I feel confident in utilizing online health information that helps to make health decisions |  |  |  |  |  |
| I can develop new knowledge based on existing health information |  |  |  |  |  |
| I can identify the purpose of a  health website |  |  |  |  |  |
| Exploring health-related information on the Internet will expand my knowledge in this field |  |  |  |  |  |
| I can quickly scan or skim a  health web page to get relevant information. |  |  |  |  |  |

1. **What types of sources the university students used for accessing online health information? (Check all that apply)**

| **Types of web sources for accessing online health information** | **Never** | **Rarely** | **Occasionally** | **Very**  **Often** | **Almost**  **Always** |
| --- | --- | --- | --- | --- | --- |
| WhatsApp |  |  |  |  |  |
| Twitter |  |  |  |  |  |
| Instagram |  |  |  |  |  |
| YouTube |  |  |  |  |  |
| Blogs |  |  |  |  |  |
| Facebook |  |  |  |  |  |
| Online News portals, Magazines |  |  |  |  |  |
| General search engines (e.g., Google, Bing, Yahoo!, Ask.com) |  |  |  |  |  |
| Government health Websites/ portals |  |  |  |  |  |

1. **How can university students evaluate the credible health-related information bestowed on online?**

For evaluating information credibility, I always check-

| **Health information evaluation** | **Strongly**  **disagree** | **Disagree** | **Undecided** | **Agree** | **Strongly**  **agree** |
| --- | --- | --- | --- | --- | --- |
| the up to date the health information |  |  |  |  |  |
| the objectiveness of the health information |  |  |  |  |  |
| the neutrality of the health information |  |  |  |  |  |
| the accuracy and quality of the health information |  |  |  |  |  |
| the relevancy of health information |  |  |  |  |  |
| the trustworthiness of health website |  |  |  |  |  |
| the coverage and scope of the health information |  |  |  |  |  |
| the transparency of the health information |  |  |  |  |  |

1. **What types of Challenges university students face while accessing and evaluating online health information?**

| **Challenges in accessing and evaluating health information (CAEHI)** | **Strongly**  **disagree** | **Disagree** | **Undecided** | **Agree** | **Strongly**  **agree** |
| --- | --- | --- | --- | --- | --- |
| Electricity failure is a common problem while using the device for searching health information |  |  |  |  |  |
| Low internet bandwidth |  |  |  |  |  |
| Lack of knowledge to access health information |  |  |  |  |  |
| Lack of training and instructions on health information literacy skills |  |  |  |  |  |
| Time management |  |  |  |  |  |
| I feel problem for determining the accuracy and quality of the health information |  |  |  |  |  |
| I could not verify the trustworthiness of health website |  |  |  |  |  |
| I could not understand the coverage and scope of the health information |  |  |  |  |  |
